# Transvaginal Natural orifice transluminal endoscopic hysterectomy vs. abdominal laparoscopic approach in real-life practices: a retrospective cohort study

**DOI:** 10.1007/s00404-025-08195-0

**Published:** 2025-09-24

**Authors:** Ala Aiob, Yara Nakhleh Francis, Haya Hebi, Saaed Awwad, Susana Mustafa Mikhail, Avishalom Sharon, Inshirah Sgayer, Lior Lowenstein

**Affiliations:** 1 Department of Obstetrics and Gynecology, Galilee Medical Center, 89 Nahariya-Cabri, Nahariya, Israel; 2 Azrieli Faculty of Medicine, Bar Ilan University, Safed, Israel

**Keywords:** Surgical techniques, Transluminal surgery, Vaginal surgery, Minimally invasive techniques

## Abstract

**Purpose:**

This study aims to retrospectively evaluate the surgical outcomes of total hysterectomy performed via Vaginal Natural Orifice Transluminal Endoscopic Surgery (vNOTES) compared to laparoscopic hysterectomy (LAP) in treating benign gynecologic conditions in a real-world clinical setting.

**Methods:**

This retrospective cohort study was conducted at Galilee Medical Center, Israel, between March 2021 and 2023. A total of 133 women were included in the study. The vNOTES group comprised 58 women, while 75 women underwent LAP. All women eligible for laparoscopic hysterectomy for benign conditions were offered the vNOTES approach. Exclusion criteria included prior pelvic radiation, active genital infections, rectovaginal endometriosis, or the need for oncogynecologic surgery related to malignancy.

**Results:**

The vNOTES group demonstrated significantly shorter operative times (69.5 vs. 117.4 min, p < 0.001) and anesthesia durations (102 vs. 146.3 min, p < 0.001), as well as reduced blood loss (108.6 mL vs. 237.9 mL, p < 0.001) compared to the LAP group. Postoperative pain, assessed by VAS scores, was significantly lower in the vNOTES group than in the LAP group (2.33 vs. 3.11, p = 0.013), with fewer requests for intravenous analgesics. No significant differences were observed between the groups in complication rates, conversion to laparotomy, or postoperative hospital stays.

**Conclusion:**

The vNOTES hysterectomy demonstrated shorter operative and anesthesia times, reduced blood loss, lower postoperative pain, and intravenous analgesic requirements compared to laparoscopic hysterectomy, without increasing complication rates. These findings suggest that vNOTES offers a viable, minimally invasive alternative to conventional laparoscopic hysterectomy for the treatment of benign gynecologic conditions. Further prospective studies are needed to assess long-term outcomes and the impact on quality of life.

## Take-home message

**Table:** 

vNOTES hysterectomy, a safe and effective minimally invasive alternative to conventional laparoscopic hysterectomy for benign gynecologic conditions, provides significant advantages. These advantages include shorter operative and anesthesia times, reduced blood loss, and less postoperative pain without increasing complication rates. The safety of vNOTES supports its growing use in appropriate clinical settings.	

## Introduction

The origins of vaginal hysterectomy can be traced back to 1512, when an Italian surgeon performed the procedure to treat uterine prolapse [1]. Despite challenges such as difficulties in managing adhesions, limited pelvic visualization, and technical obstacles in women without uterine prolapse, vaginal hysterectomy remains the preferred method, as supported by evidence from the Cochrane Library [2, 3]. Historically, laparoscopy has been favored for hysterectomy due to its reduced morbidity compared to laparotomy. Feasibility studies indicate that vaginal hysterectomy for gynecological surgeries is a viable option in over 60% of cases for benign indications, with vaginal hysterectomy preferred for uterine fibroids when the patient’s health permits, even in women without a history of prior vaginal delivery [4–6].

A novel approach, Natural Orifice Transluminal Endoscopic Surgery (NOTES), integrates the benefits of vaginal hysterectomy and laparoscopy, facilitating incision-free surgery with quicker healing and recovery, reduced postoperative pain, fewer incision site infections, and better cosmetic results while maintaining excellent visualization of the pelvic cavity. Since the introduction of vaginal NOTES (vNOTES) in 2012, numerous reports have emerged in medical literature. vNOTES includes techniques such as vaginal hysterectomy, conducted entirely via a vaginal laparoscopic approach, and “vaginally assisted NOTES hysterectomy,” which begins with a standard vaginal technique before completing the procedure laparoscopically through the vagina [7–16]. This study aims to retrospectively compare the surgical outcomes of total hysterectomy utilizing vNOTES versus conventional laparoscopic hysterectomy in treating benign gynecologic conditions in a real-world clinical setting.

## Material and methods

The study was approved by the Galilee Medical Center Institutional Review Board [NHR-0204–21-NHR], and informed consent was waived due to its retrospective nature. This retrospective data collection involved consecutive patients who underwent vNOTES hysterectomy, with or without unilateral or bilateral oophorectomy, for benign gynecological conditions at Galilee Medical Center from March 2021 to February 2023. The control group comprised women who underwent abdominal laparoscopic hysterectomy, with or without unilateral or bilateral oophorectomy, for analogous indications during the same timeframe. Data retrieval was facilitated through electronic patient records. During this period, all women eligible for laparoscopic hysterectomy, with or without oophorectomy, for benign gynecological conditions were offered the option of vNOTES, regardless of uterine size or surgical or gynecological history (e.g., endometriosis, adhesions, prior pelvic surgeries, or cesarean sections).

Exclusion criteria included the following: pelvic radiation therapy, vaginal anatomical disorders, active infections of the lower genital tract, obliteration of the pouch of Douglas observed during clinical examination, known or suspected malignancy, rectovaginal endometriosis, a history of surgical intervention for rectovaginal endometriosis, prior rectal surgery, and previous pelvic radiotherapy [10, 17]. Women who declined to undergo vNOTES proceeded with a laparoscopic abdominal hysterectomy and were subsequently included as a control group for retrospective analysis.

During preoperative counseling, all eligible women were informed that vNOTES is a novel, minimally invasive surgical option and were given the choice between vNOTES and conventional laparoscopy. The decision was documented in the medical record as part of routine preoperative documentation. The choice of surgical technique was driven solely by patient preference, without physician recommendation or bias. However, in cases where anatomical contraindications—such as suspected obliteration of the pouch of Douglas, rectovaginal endometriosis, or prior rectal surgery—were identified during clinical assessment, vNOTES was not offered, and these patients were excluded from the overall group. This ensured that only technically feasible cases were considered for vNOTES, and these criteria were consistently applied. All vNOTES and laparoscopic abdominal hysterectomy procedures were performed by a small, consistent group of surgeons with comparable expertise in advanced minimally invasive gynecologic surgery. At least two senior surgeons were present in each procedure. To reduce variability, the same surgical teams performed both vNOTES and laparoscopic hysterectomies throughout the study period. This approach helped ensure uniform surgical standards and minimize differences attributable to operator experience.

Postoperative pain was assessed using the VAS. To address this pain, patients were offered both intravenous and oral patient-controlled analgesia. This regimen included paracetamol (1 g, up to three times daily), intravenous metamizole (1 g, up to four times daily), oral ibuprofen (400 mg, up to six times daily), and intravenous tramadol (100 mg) combined with intravenous metoclopramide (10 mg). All patients received paracetamol and metamizole upon returning to the department, in accordance with the postoperative pain management protocol. Subsequently, analgesics were provided upon request. Follow-up evaluations are conducted for two months post-surgery.

### V-Notes technique

**Fig. 1:**
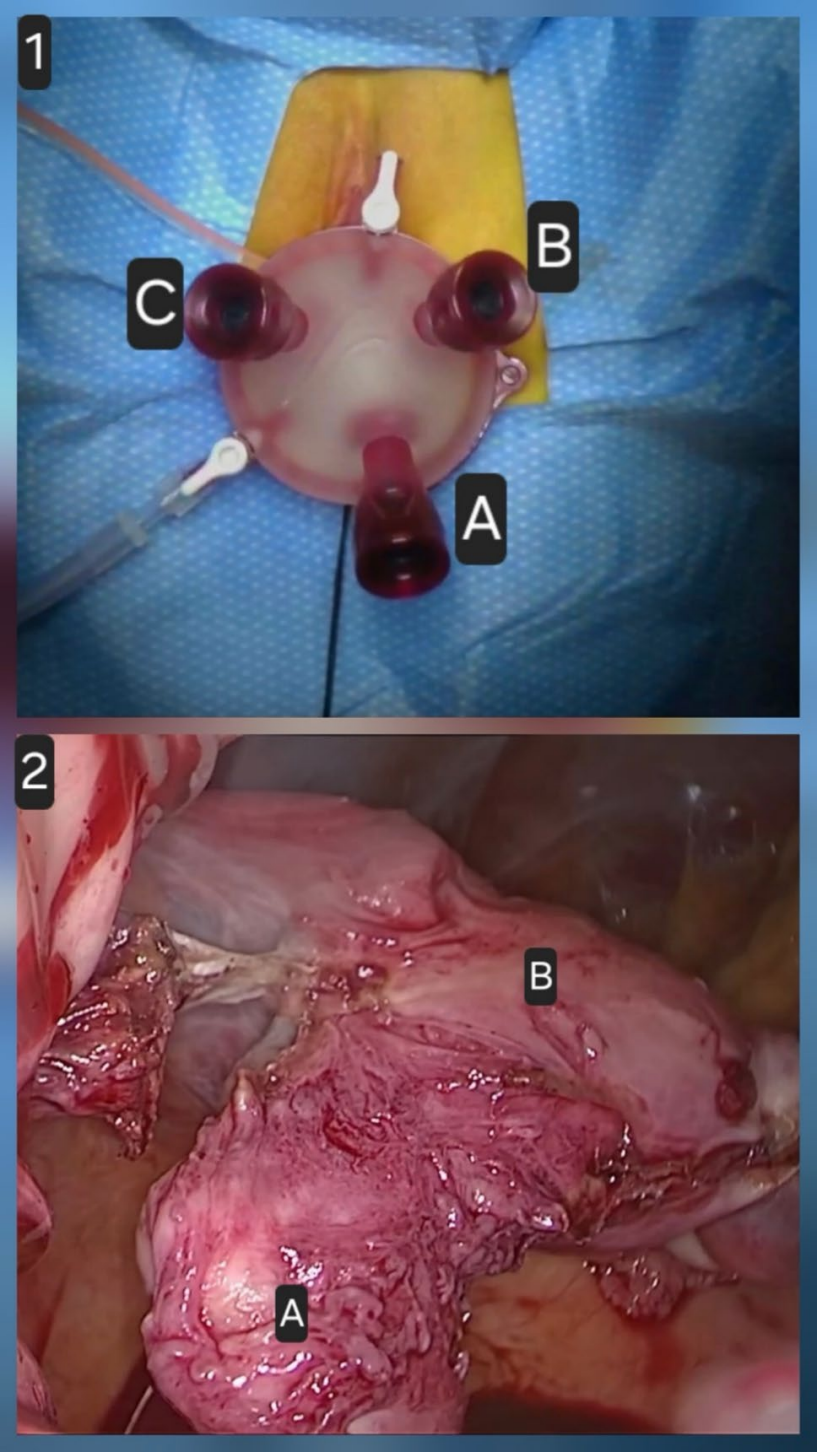
vNOTES approach. (1) Port placement: The procedure starts with cervical circumcision, followed by anterior and posterior colpotomies. After transecting the uterosacral ligaments, a multichannel GelPoint vPath port is inserted transvaginally. The endoscope is placed through the central channel **A**, while working instruments are inserted through the lateral channels **B**, **C**. The port is positioned in the vaginal fornix, providing direct access to the pelvis. (2) Intraoperative pelvic view: A 30° endoscope offers a caudal-to-cranial view. Dissection progresses in a stepwise manner: uterine arteries are skeletonized, coagulated, and transected near the uterus; the broad ligaments and round ligaments are divided; and the uterus is mobilized and then removed transvaginally; in some cases, transvaginal morcellation is necessary

The vNOTES Hysterectomy procedure utilizes a method similar to conventional Vaginal Hysterectomy (VH), starting with the dissection of the most caudal aspect of the uterus under direct visualization. The following steps of the hysterectomy are performed endoscopically while the patient is positioned in the lithotomy position. Prophylactic antibiotics, specifically cefazolin 2 g, are administered intravenously, followed by bladder evacuation facilitated by inserting a Foley catheter after proper disinfection. The procedure starts with cold-knife circumcision of the cervix, which is preceded by the infiltration of a ropivacaine-adrenaline solution for hydro-dissection, vasoconstriction, and additional local anesthesia. Cold scissors are used for both anterior and posterior colpotomy. The uterosacral ligaments are then clamped and transected using cold scissors. The vNOTES port (Gelpoint vPath) is inserted through anterior and posterior colpotomies to provide access. Carbon dioxide insufflation is carried out to establish pneumoperitoneum, achieving a maximum intraperitoneal pressure of 12 mm Hg. A 30-degree endoscope is then introduced, and the uterus is dissected from caudal to cranial using standard endoscopic instruments. A salpingo-oophorectomy is performed, if necessary, with coagulation and cutting of the infundibulopelvic ligament. Prophylactic salpingectomy is routinely conducted if the ovaries are retained, aimed at reducing the risk of epithelial ovarian cancer [18]. Morcellation is employed for large uteri that cannot be extracted vaginally. After this, the vaginal cuff is closed with a running Vicryl 1 suture, and the uterosacral ligaments are reattached under direct visualization** (**Fig. 1**).**

### Laparoscopic technique

**Fig. 2:**
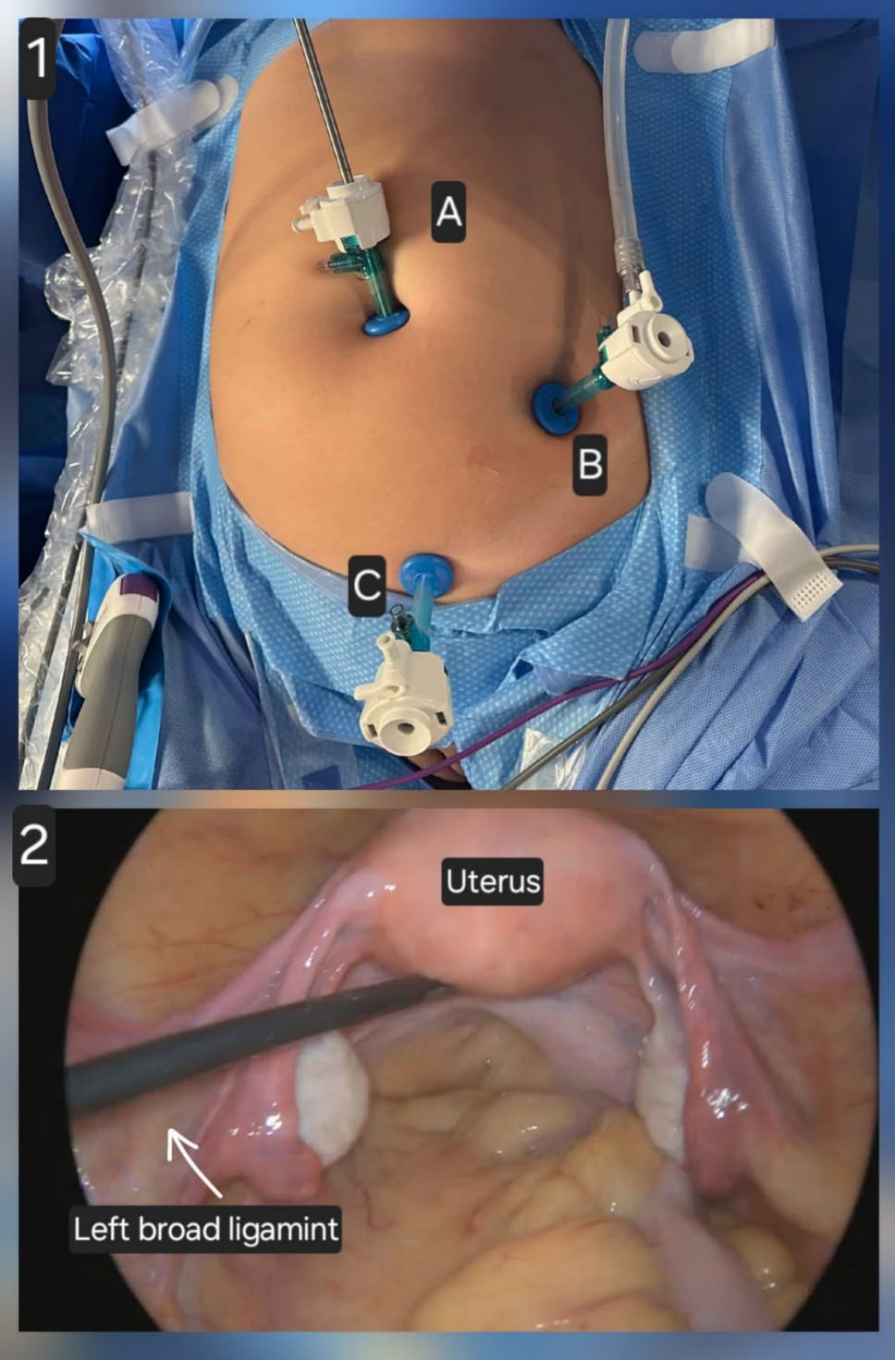
Abdominal Laparoscopic approach. (1) Port placement: Abdominal access is achieved via an umbilical trocar for the laparoscope **A**, supplemented by suprapubic and lateral trocars **B**, **C** for working instruments. The orientation of the endoscope from the anterior abdominal wall into the pelvis is shown. (2) Intraoperative pelvic view: The round ligaments are identified, coagulated, and divided. The broad ligament is opened on both sides, and the bladder is dissected away from the lower uterine segment. The uterine arteries are skeletonized, coagulated, and transected near the uterus. Circumferential colpotomy is then performed using monopolar energy. The uterus is detached and removed vaginally; in selected cases, transvaginal morcellation is required

The patient is positioned in the dorsal lithotomy position under general anesthesia, with careful padding of pressure points. A Foley catheter is inserted to decompress the bladder, and prophylactic antibiotics (e.g., cefazolin 2 g IV) are administered preoperatively. After achieving pneumoperitoneum with a Veress needle or an open (Hasson) technique at the umbilicus, a 10 mm trocar is inserted for the laparoscopic camera. Two additional ancillary trocars (5 mm) are placed under direct visualization, typically in the lower quadrants and suprapubic region. The procedure begins with an inspection of the abdominal and pelvic organs. The round ligaments are identified, coagulated, and transected using bipolar energy or an advanced vessel-sealing device. The anterior leaf of the broad ligament is opened bilaterally, and the bladder is carefully dissected off the lower uterine segment using sharp and blunt dissection. The infundibulopelvic ligaments (if the ovaries are to be removed) or the utero-ovarian ligaments (if the ovaries are to be preserved) are coagulated and transected to mobilize the adnexa. Prophylactic salpingectomy is routinely performed if the ovaries are retained, aimed at reducing the risk of epithelial ovarian cancer [18]. The uterine arteries are skeletonized, coagulated, and divided close to the uterus. Colpotomy circumferentially uses monopolar energy, a laparoscopic harmonic scalpel, or a ligature. The uterus is detached and removed vaginally or, if necessary, transvaginal morcellation is performed. The vaginal cuff is closed laparoscopically with running sutures, typically using absorbable material (Vicryl 1). After the procedure, the abdomen and pelvis are inspected for hemostasis. Ports are removed under direct visualization, the pneumoperitoneum is evacuated, and port sites are closed as necessary **(**Fig. 2**)**.

Patient characteristics recorded included age, body mass index (BMI), and surgical and obstetric history. Procedure-related variables encompassed operation time (the total duration from skin incision to wound closure), intraoperative bleeding, and the necessity for conversion to abdominal laparoscopy or laparotomy. Uterine weight was obtained from pathology reports. Additionally, intraoperative, immediate postoperative, and late postoperative complications were documented, along with hospitalization duration and postoperative requirements, as assessed by the Visual Analog Scale (VAS) for pain. Postoperative pain was evaluated using the VAS, with data from computerized nursing records; the highest value recorded on postoperative day one was kept for analysis.

### Statistical analysis

The study endpoints included operative and anesthesia times, hemoglobin levels on postoperative day 1, VAS scores, quantity of analgesic medications administered, intraoperative and postoperative complications, and length of postoperative hospital stay. Age, BMI, operative time, estimated blood loss, change in hemoglobin on postoperative day 1, and uterine weight were treated as continuous variables and are reported as mean ± standard deviation. Conversely, other variables, classified as discrete, are presented as median values with their corresponding ranges. Continuous variables exhibiting normal distributions were reported as means ± standard deviations, while median values and ranges were employed to describe variables that did not follow a normal distribution. Categorical variables were compared between groups using the Chi-square or Fisher’s exact test when expected frequencies were less than 5. Continuous variables were compared using the Mann–Whitney or independent t-test, contingent upon identifying a normal distribution. The distribution shape was primarily assessed via histogram analysis. A p-value of less than 0.05 was deemed statistically significant. Statistical analyses were conducted using IBM SPSS Statistics software, version 25.0.

## Results

A total of 133 patients who underwent hysterectomy at our center met the inclusion criteria for this study. Among these, 58 patients (43.6%) were assigned to the vNOTES group, while 75 patients (56.4%) were assigned to the LAP group. The mean age of patients in the vNOTES group was 48.6 ± 6.4 years, compared to 49.0 ± 9.1 years in the LAP group, with no statistically significant difference observed between the two groups (p = 0.197). Baseline characteristics were comparable between the two groups, including body mass index (BMI), obstetric history, the number of prior pelvic surgeries, and preoperative hemoglobin levels (Table 1).

**Table 1:** Baseline characteristics of the study population and Multivariate analysis of surgical outcomes

	VNOTES N = 58 (43.6%)	Laparoscopy N = 75 (56.4%)	P-Value
Age (year), median (range)	48.5 (33–72)	49.0 (39–90)	0.197^*^
BMI, median (range)	26.7 (18.3–39.8)	25.8 (18.4–46.8)	0.637^*^
Parity, median (range)	2.5 (0–6)	2 (0–7)	0.072^#^
Pelvic Surgery, n (%)	32 (55.2%)	51 (68.0%)	0.151^##^
Hysterectomy indications, n (%)			
Cervical dysplasia	6 (10.3%)	10 (13.3%)	0.789^##^
Menorrhagia/AUB	35 (60.3%)	47 (62.7%)	0.858^##^
Myoma	30 (51.7%)	3 (4.0%)	< .001^##^
Atypical hyperplasia	2 (3.4%)	5 (6.7%)	0.468**
Endometriosis/adenomeyosis	1 (1.7%)	8 (10.7%)	0.077^**^
HB before hysterectomy, mean(± SD)	11.8 (± 1.7)	11.5 (± 1.7)	0.392^*^
Anesthesia total time(minuets), mean(± SD)	102 (± 27.9)	146.3 (± 35.2)	<0 .001^*^
Surgery total time(minuets), mean(± SD)	69.5 (± 23.2)	117.4 (± 34.0)	< 0.001^#^
Adhesions, n (%)	5 (8.6%)	29 (38.7%)	< 0.001^##^
Uterus wight (gram), mean(± SD)	267.70 (± 201.6)	224.01 (± 151.65)	0.159^#^
Complication in the surgery, n (%)	1 (1.7%)	4 (5.3%)	0.386 ^**^
Estimated Bleeding(cc), mean(± SD)	108.6 (± 117.9)	237.9 (± 333.8)	<0 .001^#^
Post operation complications, n (%)	4 (6.9%)	7 (9.3%)	0.755^**^
Bladder perforation	2	1	
PC need	0	1	
Ileus	0	1	
Collection	0	2	
Hb after surgery, mean(± SD)	10.58 (± 1.42)	10.57(± 1.58)	0.955^*^
Analgetic treatment, n (%)	57 (98.3%)	75 (100%)	0.436^**^
IV treatment, median (range)	6 (0–16)	7 (1–24)	0.080
VAS, mean(± SD)	2.33 (± 2.00)	3.11 (± 2.09)	0.013^#^
VAS > 4, n (%)	10 (23.8%)	32 (52%)	0.004^##^
Hospitalization (days), median (range)	2 (1–4)	2 (1–9)	0.557
Hospitalization after discharge (days), n (%)	2 (3.4%)	6 (8.2%)	0.300^**^

^*^T-test; ^#^, Mann–Whitney; ^**^, Fisher; ^##^ Chi-Square

*BMI* Body Mass Index, *AUB* Abnormal Uterine Bleeding, *HB* Hemoglobin, *PC* Packed Cells, *IV* Intravenous, *VAS* Visual Analog Scale

The data are presented as number (%), mean ± standard deviation, or median (range)

Regarding the indications for hysterectomy, the vNOTES group showed a notably higher percentage of leiomyoma cases compared to the LAP group (51.7% vs. 4.0%, p < 0.001). However, no significant differences were observed for other indications, including cervical dysplasia, endometriosis, adenomyosis, abnormal uterine bleeding, hyperplasia, or uterine weight.

In terms of surgical outcomes, the mean operative time was significantly shorter in the vNOTES group (69.5 min; range 28–125) compared to the LAP group (117.4 min; range 57–213) (p < 0.001). Total anesthesia time was also decreased in the vNOTES group (102 min; range 38–162) compared to the LAP group (146.3 min; range 84–258) (p < 0.001). Additionally, the mean estimated blood loss was significantly lower in the vNOTES group (108.6 ± 117.9 mL) compared to the LAP group (237.9 ± 333.8 mL) (p < 0.001).

There were no statistically significant differences between the groups regarding the conversion to an alternative surgical route, the need for blood transfusion, reoperation, significant ileus or emesis, or the length of hospital stay. Notably, none of the patients in either group required reoperation during follow-up visits two months post-surgery.

The mean VAS score was significantly lower in the vNOTES group compared to the LAP group (2.33 ± 2 vs. 3.11 ± 2.09, p = 0.013), with a greater proportion of patients in the LAP group reporting VAS scores exceeding 4 (23.8% vs. 52%, p = 0.004). Regarding postoperative pain management, 98.3% of patients in the vNOTES group received analgesic treatment, compared to 100% of patients in the LAP group. We observed a greater need for intravenous analgesics in the laparoscopic group compared to the vNOTES group; however, this difference did not reach statistical significance (p = 0.080).

## Discussion

This study demonstrates that anesthesia and operative times were significantly shorter for vNOTES, and the technique was associated with reduced blood loss and less postoperative pain. On the first postoperative day, patients in the vNOTES group reported significantly lower VAS scores, with fewer reporting scores above 4. The shorter operative time for vNOTES (69.5 min vs. 117.4 min; p < 0.001) aligns with findings from Chaccour et al. [19], Michener et al. [20], and Park et al. [21], indicating that vNOTES may offer a more streamlined procedure, potentially due to natural orifice access and reduced trocar use. Furthermore, anesthesia time was significantly shorter in the vNOTES group (102 min vs. 146.3 min; p < 0.001), an outcome that may reduce anesthesia-related risks, especially in patients with comorbidities, and contribute to faster recovery times.

Furthermore, we observed significantly lower blood loss in the vNOTES group compared to the LAP group (108.6 mL vs. 237.9 mL; p < 0.001). However, this difference did not translate into a significant decline in hemoglobin levels or a notable decrease in postoperative hemoglobin. These findings align with Chaccour et al. [19] and Michener et al. [20], who reported no significant differences in blood loss between the two techniques. Notably, while our study documented greater estimated blood loss in the LAP group, this did not result in increased transfusion rates or significant declines in hemoglobin levels. This indicates that both techniques exhibit comparable safety profiles in managing perioperative blood loss and minimizing the risk of complications.

Patients in the vNOTES group reported significantly lower postoperative pain, as indicated by lower VAS scores on the first postoperative day (2.33 vs. 3.11; *p* = 0.013), with fewer patients experiencing VAS scores above 4 (23.8% vs. 52%; *p* = 0.004). We also observed a higher need for IV analgesics in the laparoscopy group, although this difference was marginally significant (p = 0.080). This finding is consistent with the literature, where multiple studies, including Chaccour et al. [19], Baekelandt et al. [22], and Michener et al. [20], reported lower postoperative pain and reduced analgesic requirements for vNOTES compared to laparoscopic hysterectomy. The reduced pain may be attributed to the absence of abdominal incisions and the minimally invasive nature of vNOTES, which avoids the necessity for multiple trocars and mitigates abdominal trauma. This advantage can lead to faster recovery, greater patient satisfaction, and potentially shorter hospital stays.

Contrary to findings from Baekelandt et al. [22] and Park et al. [21], which suggest shorter hospital stays for vNOTES, our study found no significant difference in discharge timing between the groups. This discrepancy may reflect variations in discharge criteria, postoperative protocols, or patient demographics. Nonetheless, vNOTES patients experienced faster recovery and reduced pain, which may facilitate a quicker return to normal activities. However, this did not affect the duration of hospital stays in this cohort.

Overall, there were no significant differences in intraoperative or postoperative complication rates between the vNOTES and laparoscopic groups, suggesting a comparable safety profile for both techniques. These findings are consistent with previous reports in the literature, which support the feasibility, safety, and advantages of vNOTES hysterectomy [23–25].

A key strength of our study is its reflection of real-world clinical practice through the inclusion of a heterogeneous patient population. Unlike studies that rely on narrowly selected cohorts, we offered vNOTES as a routine surgical option to all women eligible for standard laparoscopy, without applying additional selection criteria. Although the sample size is relatively modest, this represents one of the largest single-center cohorts in which vNOTES was implemented as part of standard care. This approach enabled a more accurate assessment of the technique’s feasibility and applicability across diverse clinical scenarios commonly encountered in gynecologic practice. Nonetheless, the retrospective, non-randomized design introduces inherent limitations, including potential selection bias and the absence of standardized discharge criteria. While all patients were consecutively enrolled and uniformly counseled, confounding factors may still exist. Future prospective studies with larger sample sizes and long-term follow-up are needed to further validate the safety, efficacy, and broader clinical utility of vNOTES.

As this study reflects real-world clinical implementation, a learning curve was naturally present. However, early cases were not excluded or stratified. All procedures were performed by surgeons already experienced in both vaginal and laparoscopic hysterectomy, and each vNOTES procedure was conducted under the supervision of a senior surgeon with specific expertise in this technique. This approach helped standardize surgical performance and mitigate potential bias related to the learning curve.

In conclusion, this study adds to the evidence supporting vNOTES as a beneficial alternative to laparoscopic hysterectomy. It offers advantages such as shorter operative and anesthesia times, reduced blood loss, and lower postoperative pain without compromising safety. As the technique matures, it holds promise for becoming a preferred minimally invasive option in select patient groups. While the findings are promising, they should be interpreted with caution due to the study’s retrospective nature and the lack of randomization. Future studies should include larger prospective cohorts with long-term follow-up and patient-centered outcomes such as postoperative quality of life and return to daily activity.

## Data Availability

No datasets were generated or analysed during the current study.
